# Parliament and the Profession

**Published:** 1917-11-10

**Authors:** 


### Parliament and the Profession.

### Welsh Discharged Soldiers.

THE Parliamentary Secretary to the Ministry of Pensions informed Major Davies that some 176 institutions, including military, auxiliary, and civil hospitals and convalescent homes, are in use and open to the treatment of discharged disabled men in Wales and Monmouthshire. Schemes for establishing an after-care colony with training for tuberculosis, and for at least one centre for orthopædic treatment, are now under consideration. Training centres will shortly be established at Wrexham and Cardiff.

### Welfare of the Blind.

THE Parliamentary Secretary to the Local Government Board stated that the report of the Inter-departmental Committee on the Welfare of the Blind is receiving the careful consideration of the Government, and it is hoped that it may be found possible before long to give effect to their recommendations.

### Nerve-Shock Sailors and Soldiers.

MR. KING called attention to the large proportion of mental specialists amongst the Scottish and Irish doctors serving on the Special Expert Boards which estimate the disability of ex-soldiers invalided through nerve strain. Sir A. Griffith-Boscawen replied that of the thirteen Scottish and Irish doctors serving three hold posts connected with lunacy, and expressed the opinion that a special knowledge of mental disorders does not render a medical man unsuitable to exercise the functions required.

### Soldiers Disabled by Tuberculosis and Rheumatism.

SIR A. GRIFFITH-BOSCAWEN informed Mr. Anderson that War Pension Committees have full powers to make arrangements with any existing institutions for the treatment of rheumatism, and those powers have been widely used. Treatment for tuberculosis is provided by the National Health Insurance Commissioners in conjunction with the local health authorities. The Ministry of Pensions are at present considering the question of providing after-care colonies where treatment and training may be given in cases of tuberculosis in its early stages.

### Medical Staffs of Territorial Hospitals.

MR. MACPHERSON supplied Colonel Gretton with the following particulars with regard to the medical staffs of general hospitals of the Territorial Force:

| | Beds | Medical Officers | Including | |
|---|---|---|---|---|
| 1st London ... | 1,021 | 21 | 4 lieut.-cols. and | 4 majors |
| 2nd London ... | 1,512 | 21 | 2 ,, | 5 ,, |
| 3rd London ... | 2,400 | 36 | 2 ,, | 5 ,, |
| 4th London ... | 2,171 | 31 | 2 ,, | 3 ,, |
| 5th London ... | 662 | 16 | 1 ,, | 1 ,, |

### One-Eyed Soldiers.

MR. MACPHERSON informed Mr. Watt that men with one eye are not now being passed for any category of personal service. At the present time men who have lost the sight of one eye, if otherwise fit, are passed only for the auxiliary services of the Army.

### Payments to Voluntary Hospitals.

SIR WILLIAM COLLINS asked the Financial Secretary to the War Office whether, in view of increased cost, the amount of grants-in-aid to voluntary hospitals in respect of the treatment of wounded soldiers could be increased, and was informed that the point had been under consideration for some time, and an announcement would shortly be made.

### Disabled Nurses.

THE Parliamentary Secretary to the Ministry of Pensions told Major Chapple that he had no doubt that the promoters of the fund for the endowment of the College of Nursing and the benevolent fund for nurses would take into consideration the provision made by the State for nurses disabled through war service, as set out in the Royal Warrant of August 1 last, when deciding how their funds may best be applied.

